# Thoughts on detecting tissue distribution of potential COVID-19 receptors

**DOI:** 10.2217/fvl-2020-0136

**Published:** 2020-08-12

**Authors:** Hongtao Tang, Xiaosheng Lu, Shuyan Qie, Jianing Xi

**Affiliations:** 1 ^1^Plastic Surgery Hospital, Peking Union Medical College, Chinese Academy of Medical Sciences, Beijing, China; 2 ^2^Department of Plastic Surgery, Affiliated Hospital of Weifang Medical University, Shandong, China; 3 ^3^Department of Rehabilitation, Beijing Rehabilitation Hospital affiliated to Capital Medical University, Beijing, China

**Keywords:** COVID-19, high-risk organ, receptor, RNA-seq, tissue distribution

## Abstract

**Aim:** As a novel coronavirus, coronavirus disease 2019 (COVID-19) has caused a global epidemic. Many clinical data show that COVID-19 can not only cause viral pneumonia but also damage a variety of target organs. **Materials & methods:** We searched some open online datasets, such as Gene ORGANizer, the Human Protein Atlas and Pubmed, to explore the tissue distribution of potential COVID-19 receptors (ACE2, CD209, CLEC4M and BSG) in the human body. **Results:** The above potential COVID-19 receptors were highly expressed in the lungs, intestine, kidney, liver, heart, testis, placenta, hematopoietic tissue and nerve tissue. **Conclusion:** It is speculated that they may be potentially high-risk organs susceptible to COVID-19 infection. It is expectant to provide some help for further research in the future.

## COVID-19

With the discovery of the spread of novel coronavirus in Wuhan, China in December 2019, WHO focused its attention and listed the epidemic as a ‘public health emergency of international concern’ on 30 January 2020 and officially named it as ‘COVID-19’ on February 11 [1]. Then, on 11 March, it was assessed that COVID-19 has the characteristics of a global pandemic and was a major threat to global public health [2]. As of 31 March, WHO statistics show that a total of 697, 244 cases have been confirmed worldwide [3]. In the published systematic retrospective analysis of the virus, such as Rodriguez-Morales* et al.* [4] and Fang* et al.* [5], 660 and 72 research articles respectively showed that the main organ involved in the virus was the lungs, which is characterized by symptoms of viral pneumonia, such as fever, cough, dyspnea, expectoration, fatigue, muscle soreness and so on. The common complication that happened in severe patients is acute respiratory distress syndrome, while a few of them have acute renal injury and heart injury. According to the abnormalities of routine laboratory examination of critically ill patients, some researchers think that COVID-19 infection may have the possibility of causing liver injury, immunodeficiency and activation of blood coagulation [6]. It has been found that COVID-19 is a positive-strand RNA virus with many variants and mutation sites [7]. Whether these variants will change the infectivity and pathogenicity of the virus is worthy of further discussion and research. Some research found that the D614G mutation, a change in COVID-19 spike protein, will increase its infectivity and transmissibility 2.4-times than before [8]. Therefore, when exploring the complications caused by COVID-19, we will focus on the viral receptors which attack human host cells. The distribution of these receptors in human tissues can predict the complications caused by the virus and provide early warnings for the potentially affected organs. In addition to the fact that ACE2 is known to be a receptor for COVID-19 to invade the human body, given the high homology between the virus and severe acute respiratory syndrome coronavirus (SARS-CoV), the other two SARS-CoV binding receptors *DC-SIGN* and *L-SIGN* in host cells may also be potential receptors of COVID-19 [9]. Another study has shown that COVID-19 can invade the human body through a new pathway of *CD147* receptors (also known as *BSG*, *Basigin*, *EMMPRIN*) on host cells [10].

## Gene ORGANizer

Gene ORGANizer (http://geneORGANizer.huji.ac.il/) is a kind of phenotypic tool, which directly links human genes to the organ systems they affect. It uses the two largest databases of human genetic diseases and phenotypic associations: human phenotypic ontology and DisGeNET, to establish more than 150,000 gene-organ associations and link more than 7000 genes to more than 150 anatomical sites [11]. For example, we know that mutations in the *HOXA2* gene can lead to congenital microtia with dysplasia of the external ear (OMIM ID: 612290) [12]. By using this tool to search, we can relate *HOXA2* to the following organ systems: external ear, ear, head, body surface system, head and neck regions and ectoderm.

In this article, we will detect the tissues distribution of potential COVID-19 receptors (ACE2, DC-SIGN, L-SIGN and CD147; the genotypes are ACE2, CD209, CLEC4M and BSG; Gene ID are 59272, 30835, 10332 and 682 respectively) in the Gene ORGANizer, the Human Protein Atlas (HPA) and Pubmed Gene database and compare these outputs. On the one hand, the purpose is to test the effectiveness of the application of the tool ‘Gene ORGANizer’ in the relationship between genes and organs. On the other hand, it is expected to provide early warning signals for the potentially involved organs of COVID-19 to a certain extent and help clinicians to better understand and deal with the related complications of COVID-19.

## Materials & methods

Gene ORGANizer can be retrieved in two ways: ‘browse’ and ‘ORGANize’. ‘Browse’ allows you to see the organ systems associated with a particular gene. ‘ORGANize’ can get the results of the enrichment or depletion of a list of genes expressed in the organ systems. Furthermore, ‘Confident’ represents that the association is derived from human phenotypic ontology and DisGeNET2 databases; ‘Tentative’ means that this association is derived from one of the databases or based on observations of nonhuman organisms, such as rats and mice. ‘Typical’ represents the phenotype that happened in more than 50% of the patients with the disease [11].

This time, we only need to use ‘Browse’ in this tool to retrieve the result of a single gene. Enter four potential receptor genes ‘*ACE2*, *CD209*, *CLEC4M* and *BSG*’ or Gene ID respectively in the ‘Browse’ column of the Gene ORGANizer (http://geneORGANizer.huji.ac.il/browse/). Then select the ‘organ’ item in the returned outputs, to get the results of related organs in the database.

The tissue atlas in the Human Protein Atlas (www.proteinatlas.org/) shows the expression and localization of human proteins across tissues and organs, based on deep sequencing of RNA (RNA-seq) from 37 major different normal tissue types and immunohistochemistry on tissue microarrays containing 44 different tissue types. Its’ RNA expression overview shows RNA-data from three different sources, respectively: internally generated HPA RNA-seq data, RNA-seq data from the Genotype-Tissue Expression (GTEx) project and CAGE data from FANTOM5 project, as well as the consensus data set which is based on a combination of all three sources [13].

Pubmed Gene database (www.ncbi.nlm.nih.gov/gene/) contains the gene expression in *Homo sapiens* (human) from the project named HPA RNA-seq normal tissues. They were performed of tissue samples from 95 human individuals representing 27 different tissues, to determine tissue-specificity of all protein-coding genes.

## Result

### Gene ORGANizer data

**Figure 1.**
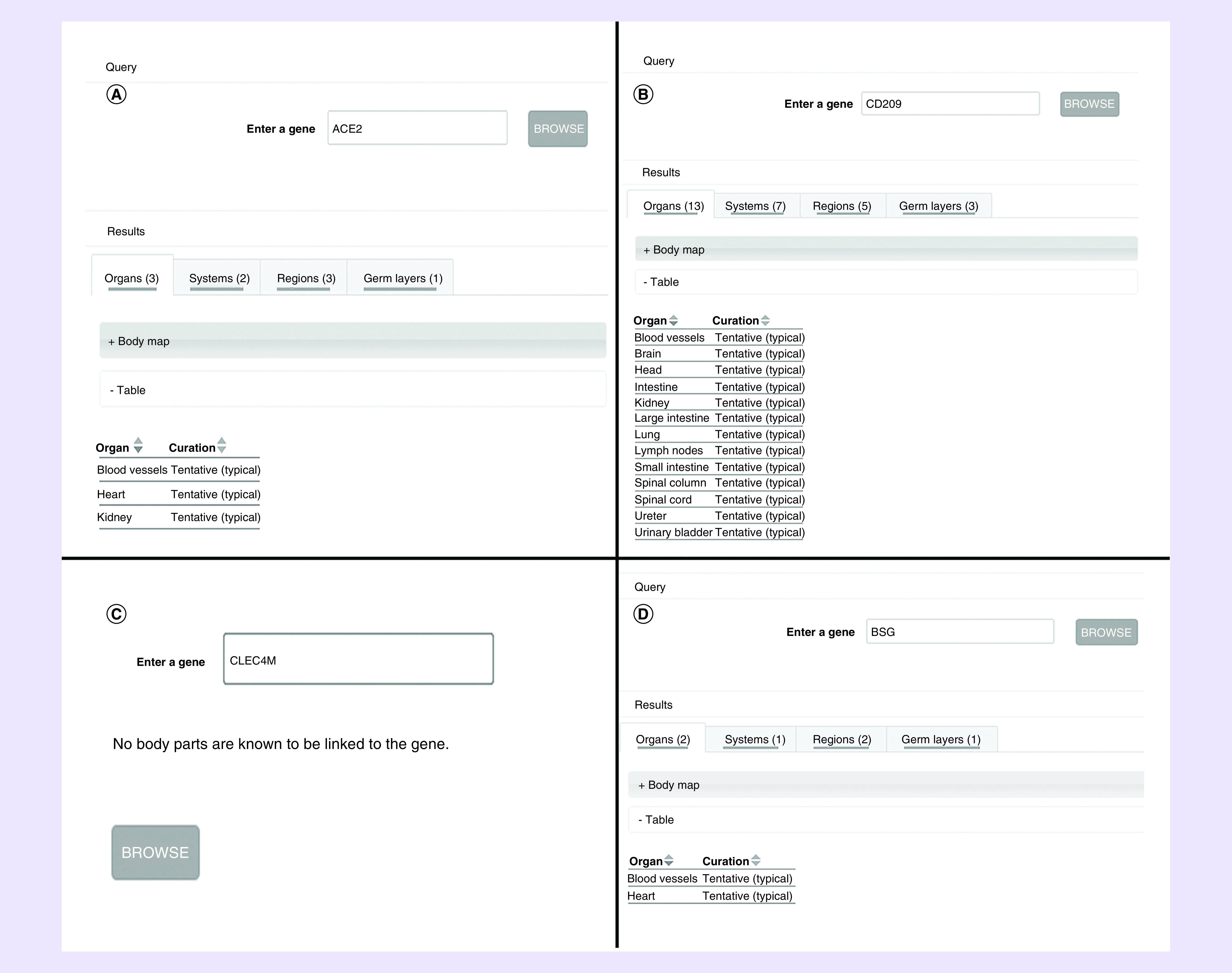
Gene ORGANizer: genes and related organs. The common organs related to *ACE2*, **(A)** *CD209*, **(B)** *CLEC4M* **(C)** and *BSG* **(D)** are blood vessels, heart and kidney.

In the results of Gene ORGANizer (Figure 1), we find that three organs ‘blood vessels, heart and kidney’ are associated with *ACE2* (Figure 1A), while *CD209* has 13 linked organs ‘blood vessels, brain, head, intestine, kidney, large intestine, lungs, lymph nodes, small intestine, spinal column, spinal cord, ureter and urinary bladder’ (Figure 1B). *BSG* is bound with ‘blood vessels and heart’ (Figure 1D), but there are no organs linked to *CLEC4M* (Figure 1C).

### The HPA & Pubmed data

**Figure 2.**
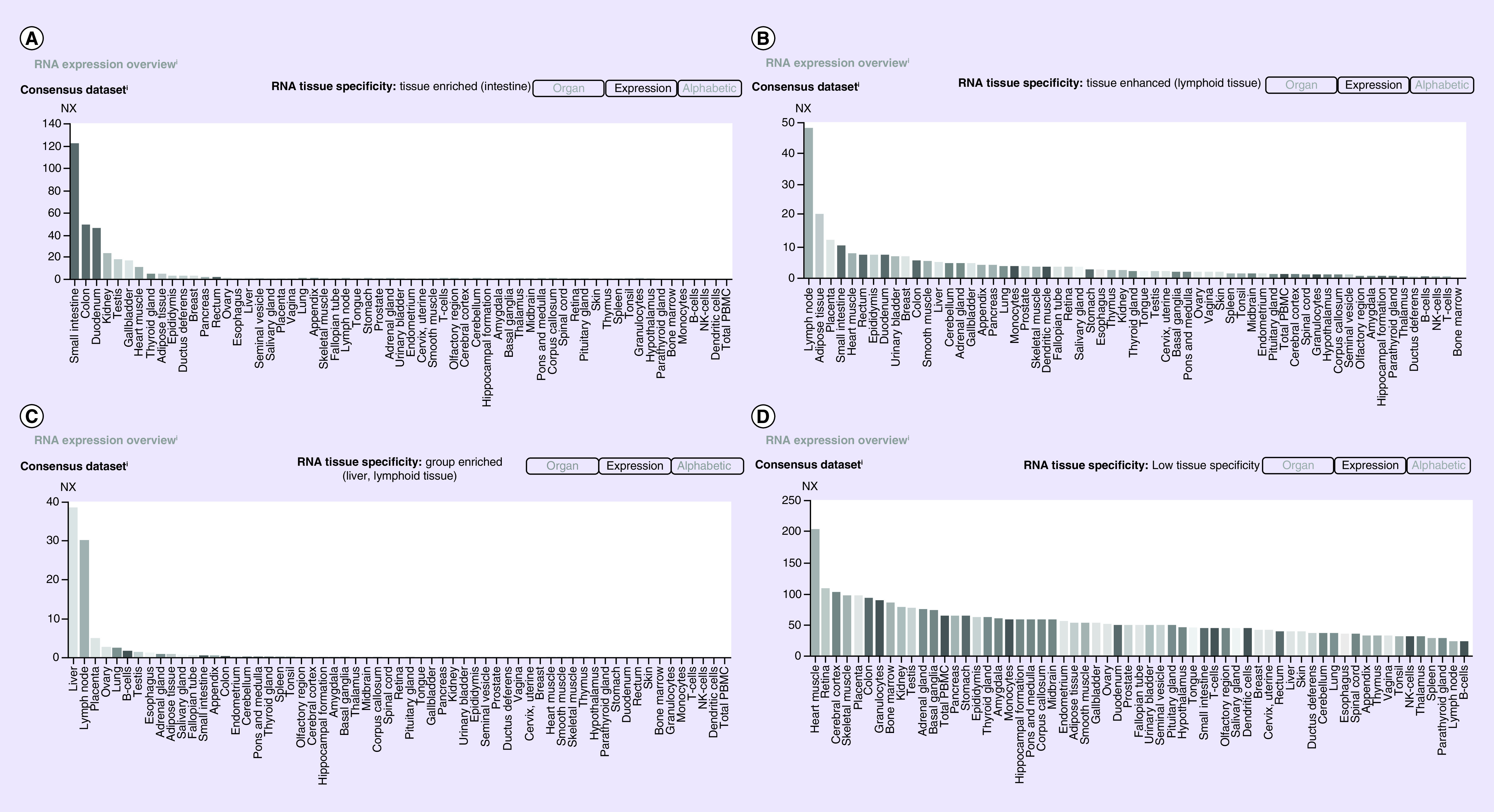
The Human Protein Atlas: the tissues expression of genes. **(A)** *ACE2* is more expressed in intestine, kidney, testis and heart than other tissues. **(B)** *CD209* is enriched in liver and lymph node. **(C)** The enriched tissue in *CLEC4M* is lymphoid tissue. **(D)** There is low tissue specificity in *BSG* and it exists widely in almost all human tissues.

**Figure 3.**
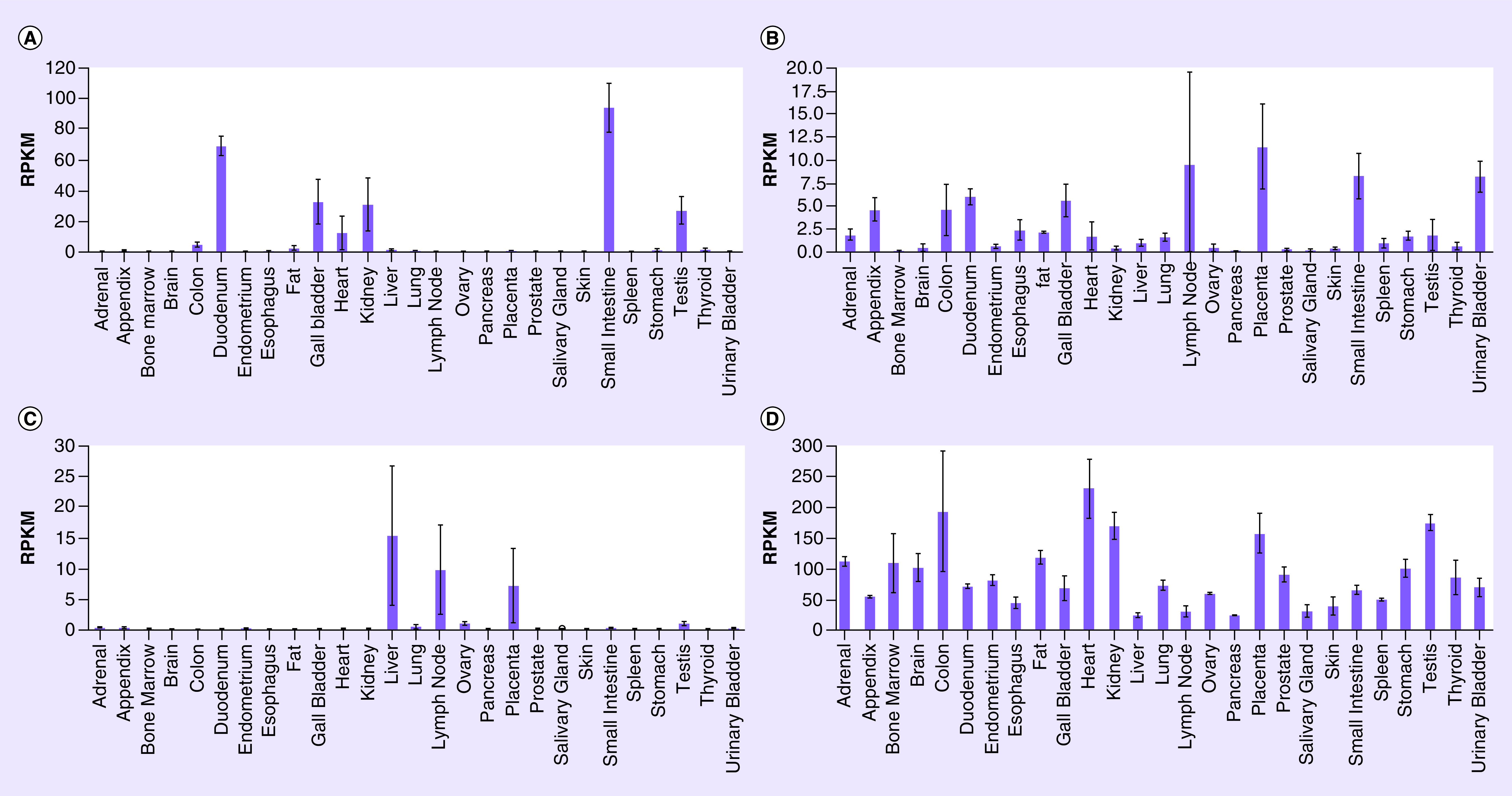
Pubmed Gene: distribution maps of gene expression in normal tissues. The genes *ACE2*, **(A)** *CD209*, **(B)** *CLEC4M* **(C)** and *BSG* **(D)** are co-expressed in the lungs, liver, placenta and testis. It shows the similar results with the HPA. HPA: Human Protein Atlas.

According to the results obtained in the HPA and Pubmed Gene database, Figure 2 shows the similar tissues distribution of these genes in the human body, as shown in Figure 3 respectively. *ACE2* is expressed in many organs, especially the small intestine and duodenum, followed by kidney, testis, gall bladder, heart, adipose tissue, epididymis, liver and other organs, while a trace distribution is also found in the lung tissue (Figures 2A & 3A). *CD209* is distributed in most human organs, including lymph nodes, adipose tissue, placenta, small intestine, heart, epididymis, duodenum, urinary bladder, liver, cerebellum, etc (Figures 2B & 3B). The expression of *CLEC4M* is mainly concentrated in the liver, lymph nodes, placenta, ovary, lungs and testis (Figures 2C & 3C). The tissue specificity of *BSG* is low and it is expressed in almost all human tissues, especially in the heart, cerebral cortex, placenta, colon, testis and kidney (Figures 2D & 3D).

In previous studies on these genes, the distribution of the above four receptors in human organs and tissues has been supplemented. ACE2 is highly expressed in alveolar epithelial cells, intestinal enterocytes, vascular endothelial cells and smooth muscle cells. It is expressed more in renal tubular epithelial cells than in glomeruli and also exists in the nasal cavity, oral mucosa and nasopharynx [14], endocardial cells and cardiomyocytes [15]. *CD209* is enriched on the surface of dendritic cells located in the thymus, tonsil, spleen and lymph nodes, followed by mucosal surface and dermis, as well as some specific macrophages, such as Hofbauer cells in the placenta, Kupffer cells in hepatic sinusoids and alveolar macrophages [16,17]. *CLEC4M* is mainly located in lymph nodes, placental capillaries, hepatic sinusoids, endothelial cells of the lungs [16], alveolar cells, capillaries in lamina propria of ileal villi and intestinal Peyer collecting lymph nodes [17]. *BSG* is present on the surface of epithelial cells, endothelial cells, hematopoietic cells (monocytes, granulocytes, T lymphocytes, erythrocytes and platelets) and tumor cells in most organs and it is highly expressed in heart, skeletal muscle, kidney, testis, thymus, bone marrow, fetal liver and lungs [18,19].

Parts of them are summarized in the following Table 1.

**Table 1.** The summary of the tissues distribution of four COVID-19 potential receptors from the results in Gene ORGANizer, the Human Protein Atlas and Pubmed.

COVID-19 potential receptors	Gene ORGANizer	HPA and Pubmed	Partial literature
ACE2	Blood vessels, heart, kidney	Small intestine, duodenum, kidney, testis, gall bladder, heart, adipose tissue, epididymis, liver, lungs, etc	Lungs, small intestine, vascular endothelial cells, smooth muscle cells, kidney, oral and nasal mucosa, heart
CD209	Blood vessels, brain, head, intestine, kidney, large intestine, lungs, lymph nodes, small intestine, spinal column, spinal cord, ureter and urinary bladder	Lymph nodes, adipose tissue, placenta, small intestine, heart, epididymis, duodenum, urinary bladder, liver, cerebellum, etc	Dendritic cells (thymus, tonsil, spleen, lymph nodes), mucosa, dermis, macrophages (placenta, hepatic sinusoids, alveoli)
CLEC4M		Liver, lymph nodes, placenta, ovary, lungs, testis	Lymph nodes, placenta, hepatic sinusoids, lungs, intestines
BSG	Blood vessels, heart	Almost all organs such as heart, cerebral cortex, placenta, colon, testis, kidney, etc	Epithelial cells, endothelial cells, hematopoietic cells, tumor cells, heart, skeletal muscle, kidney, testis, thymus, bone marrow, fetal liver, lungs

HPA: Human Protein Atlas.

## Discussion

### Potentially involved organs

As we all know, RNA-seq could convert the sample of mRNA into cDNA and sequence on a high-throughput platform. The primary data obtained from RNA-seq are mapped, assembled into expression summaries and normalized. Then, we can get the statistical test of differential expression [20]. In this article, we use the existing public RNA-seq expression data of potential COVID-19 receptors genes and obtain their specific tissue distribution according to different standards of three databases. These standards can be found on their online websites. It is precisely because of these RNA-seq data of COVID-19 that we could speculate on the results of these potentially involved organs.

According to the above results, four potential invasive receptor genes (*ACE2*, *CD209*, *CLEC4M* and *BSG*) of COVID-19 are co-expressed in the lungs, liver, placenta and testis. Except for *CLEC4M*, the receptor *L-SIGN*, the other three genes are expressed in the heart, intestine, kidney, gall bladder, blood vessels and partly in hematopoietic cells and brain tissue. Among these four genes, we pay attention to the distribution of ACE2, which has been identified as an aggressive receptor. From these results, we could speculate that the potentially high-risk organs which are vulnerable to COVID-19 infection may be lungs, intestine, kidney, testis, heart, liver, placenta, hematopoietic tissue and nerve tissue.

Referring to the clinical characteristics of existing COVID-19 cases, Fang *et al.* [5] systematic retrospective study data included 3470 patients, ‘their most common clinical symptoms were fever (83%), cough (61%), fatigue (37.9%), increased sputum (28.7%), dyspnea (14.5%)’, all of which were consistent with the manifestations of viral pneumonia. Second, ‘there were fewer patients with gastrointestinal symptoms, such as anorexia, nausea and vomiting (8.9%) and diarrhea (6.1%)’. Their common complications included acute respiratory distress syndrome (ARDS; 8.9%) and acute renal failure (2.1%). According to the fact that COVID-19 can be transmitted through the respiratory tract and digestive tract, these findings all indicate that COVID-19 has the ability to invade the lungs, intestines and kidney, which is consistent with our results. In addition, studies on the relationship between COVID-19 and renal function show that it is common for patients to have elevated levels of proteinuria, plasma creatinine and blood urea nitrogen [21]. In another study by Guan *et al.* [22], laboratory indicators such as LDH (41.0%), creatine kinase (13.7%), ALT (21.3%) and AST (22.2%), may indirectly indicate the damage of heart and liver after the virus invasion. However, based on the distribution of receptors in endocardial cells, cardiomyocytes and vascular endothelial cells of the heart, it is speculated that if the virus could attack the heart, it may cause the degeneration and necrosis of cardiomyocytes, exfoliation of endothelial cells, subendocardial ischemia and coronary atherosclerosis, leading to cardiac dysfunction and even cardiovascular diseases.

Apart from the large amount of clinical data mentioned above, in other aspects, although the distribution of these potential receptors is also abundant in testis, placenta and hematopoietic tissue, little attention has been paid to whether COVID-19 will cause unknown damage to these organs or affect blood safety and invade the nervous system.

Concerning the testis, we wonder if the virus has the opportunity to directly attack the testis through its receptors or produce a secondary autoimmune response, which leads to viral orchitis causing swelling, fever, pain and other local inflammatory manifestations. Then it may be possible to cause sexual dysfunction, male infertility and even testicular tumors in the long run [23]. However, these symptoms and diseases are often ignored by the patients and may not be diagnosed at the correct time of their infection. Or the results can only be obtained through the researches of a small number of pathological specimens. Only in a pathological study in 2006, orchitis was confirmed as one of the SARS-related complications. By observing the autopsy specimens of the testis from six patients with SARS, the team found that SARS’s infection would cause the infiltration of immune cells and cytokines in testis, thus destroying germ cells and causing orchitis [24]. Second, previous studies on the distribution of ACE2 in testis, through HPA, GTExportal [25], Gene Expression Omnibus and Sequence Read Archive [26], have confirmed that ACE2 receptors are abundantly expressed in testicular spermatogonia, Leydig cells and Sertoli cells. In accordance with us, it is speculated that the testis may be a potential target for COVID-19 infection, and if the virus could attack the spermatogenic cells of the testis, then there is a possibility that sexual transmission could also be achieved.

In terms of special groups like pregnant women, if COVID-19 can invade the placenta through its potential receptors, it will affect the placental development of these pregnant women, resulting in adverse pregnancy outcomes such as poor placental position, premature rupture of membranes, fetal growth restriction, preterm delivery, neonatal infection, perinatal death and so on [27]. However, there are few clinical data in this field and only a few studies have failed to find the potential vertical transmission of COVID-19 and the possibility of causing the above-mentioned potential events, such as Chen’s analysis [28] of the characteristics of nine infected pregnant women in Wuhan area, the Wei’s team’s research [29] on maternal and neonatal outcomes and Huang’s study [30] on placental pathology. Nevertheless, considering the particularity of pregnant women and newborns, pregnant women are more susceptible to be infected with respiratory pathogens in physiology. For that reason, they should be regarded as key groups for the prevention and management of COVID-19 [31].

Furthermore, in the view of the expression of receptors on hematopoietic cells, if COVID-19 could affect the blood system or its components fall off into the blood, it may induce the contamination of blood products and lead to limited blood transmission. Some studies have shown that the viral RNA could be detected by real-time PCR in the plasma, called RNAaemia [32]. Although this does not represent the infectious virus in the blood, it is still worthy of our vigilance. It has been reported that only one case of aplastic anemia was infected by receiving blood products from COVID-19 patients [33]. In addition, with the emergence of more and more asymptomatic patients, there is also the increasing possibility of the potential spread by their blood products [34]. Therefore, it is necessary to strengthen the detection of blood donation and the management of blood products among residents at high-risk of COVID-19. Besides, there is no definite confirmation as to whether COVID-19 could invade the nervous system. Only studies have shown that β-CoVs, such as SARS-CoV and MERS-CoV, which is homologous to COVID-19, has the ability of neurophagia [35]. And some critically ill patients have manifestations of acute cerebrovascular disease and disturbance of consciousness, while a few have symptoms of the peripheral nervous system such as hypogeusia and hyposmia [36].

In summary, although we have obtained the potential high-risk organs susceptible to COVID-19 infection by exploring the tissue distribution of potential COVID-19 receptors in the human body and summarizing the existing studies, it still needs further verification and correction through clinical practice and pathological studies. We expect that our findings could provide some help for further research in the future.

### The limitation of Gene ORGANizer

By comparing the results retrieved by Gene ORGANizer with those in the HPA and Pubmed, we realize that they are quite different and the results of this tool are not comprehensive. It is supposed that there may be the following reasons. First of all, the diseases involved in the tool are mainly hereditary originated from the two databases. It is based on these genetic diseases or phenotypes to associate genes with the organs they affect. When a gene is normally or abnormally expressed in an organ, if there is no known occurrence of genetic diseases or phenotypes, the association between the gene and the organ will not be reflected in this tool. Besides, the database version of the tool ends in September 2016. It comes naturally that the genetic diseases or phenotypes discovered after that time, as well as genes and their affected organs, will not be updated in the search results of the tool. Therefore, when we need to find out the relationship between genes and their organs, that is, the expression of genes in organs, the utilization of this tool is quite limited. This tool is a useful tool for us only when we need to study the relationship among genes, genetic diseases/phenotypes and organs.

## Conclusion

Through these open online datasets, we obtained the result that the potential COVID-19 receptors are highly expressed in the lungs, intestine, kidney, liver, heart, testis, placenta, hematopoietic tissue and nerve tissue. It is speculated that they may be potentially high-risk organs susceptible to COVID-19 infection. It is expectant to provide some help for further research in the future.

**Summary points**

- A novel coronavirus named COVID-19 has caused a global epidemic.
- Many clinical data show that COVID-19 can not only cause viral pneumonia but also damage a variety of target organs.
- We searched Gene ORGANizer, the Human Protein Atlas and Pubmed, to explore the tissue distribution of potential COVID-19 receptors (ACE2, CD209, CLEC4M and BSG).
- The potential COVID-19 receptors are highly expressed in the lungs, intestine, kidney, liver, heart, testis, placenta, hematopoietic tissue and nerve tissue.
- It is speculated that they may be potentially high-risk organs susceptible to COVID-19 infection.
